# Reactogenicity within the first week after Sinopharm, Sputnik V, AZD1222, and COVIran Barekat vaccines: findings from the Iranian active vaccine surveillance system

**DOI:** 10.1186/s12879-023-08103-4

**Published:** 2023-03-10

**Authors:** Mostafa Enayatrad, Sepideh Mahdavi, Roqayeh Aliyari, Sajad Sahab-Negah, Sairan Nili, Mohammad Fereidouni, Parvin Mangolian Shahrbabaki, Alireza Ansari-Moghaddam, Abtin Heidarzadeh, Fariba Shahraki-Sanavi, Mansooreh Fateh, Hamidreza Khajeha, Zahra Emamian, Elahe Behmanesh, Hossein Sheibani, Maryam Abbaszadeh, Reza Jafari, Maryam Valikhani, Ehsan Binesh, Hamid Vahedi, Reza Chaman, Hamid Sharifi, Mohammad Hassan Emamian

**Affiliations:** 1 Clinical Research Development Unit, Bahar Hospital, Shahroud University of Medical Science, Shahroud, Iran; 2 Department of Epidemiology, School of Public Health, Shahroud University of Medical Sciences, Shahroud, Iran; 3 Neuroscience Research Center, Mashhad University of Medical Sciences, Mashhad, Iran; 4 Department of Public Health, Faculty of Health, Kurdistan University of Medical Sciences, Sanandaj, Iran; 5 Cellular and Molecular Research Center, Birjand University of Medical Sciences, Birjand, Iran; 6 Department of Critical Care, Razi Faculty of Nursing and Midwifery, Nursing Research Center, Kerman University of Medical Sciences, Kerman, Iran; 7 Health Promotion Research Center, Zahedan University of Medical Sciences, Zahedan, Iran; 8 School of Medicine, Guilan University of Medical Sciences, Rasht, Iran; 9 Infectious Diseases and Tropical Medicine Research Center, Zahedan University of Medical Sciences, Zahedan, Iran; 10 Center for Health Related Social and Behavioral Sciences Research, Shahroud University of Medical Sciences, Shahroud, Iran; 11 Ophthalmic Epidemiology Research Center, Shahroud University of Medical Sciences, Shahroud, Iran; 12 Health Technology Incubator Center, Shahroud University of Medical Sciences, Shahroud, Iran; 13 Clinical Research Development Unit, Imam Hossein Hospital, Shahroud University of Medical Science, Shahroud, Iran; 14 School of Allied Medical Sciences, Shahroud University of Medical Sciences, Shahroud, Iran; 15 HIV/STI Surveillance Research Center, and WHO Collaborating Center for HIV Surveillance, Institute for Futures Studies in Health, Kerman University of Medical Sciences, Kerman, Iran

**Keywords:** Vaccine reactogenicity, Sinopharm, Sputnik V, AZD1222, COVIran Barekat

## Abstract

**Background:**

This study aimed to evaluate the reactogenicity effects of COVID-19 vaccines, used in Iran.

**Methods:**

At least 1000 people were followed up with phone calls or self-report in a mobile application within 7 days after vaccination. Local and systemic reactogenicities were reported overall and by subgroups.

**Results:**

The presence of one or more local and systemic adverse effects after the first dose of vaccines was 58.9% [(95% Confidence Intervals): 57.5–60.3)] and 60.5% (59.1–61.9), respectively. These rates were reduced to 53.8% (51.2–55.0) and 50.8% (48.8–52.7) for the second dose. The most common local adverse effect reported for all vaccines was pain in the injection site. During the first week after the first dose of vaccines, the frequency of the pain for Sinopharm, AZD1222, Sputnik V, and Barekat was 35.5%, 86.0%, 77.6%, and 30.9%, respectively. The same rates after the second dose were 27.3%, 66.5%, 63.9%, and 49.0%. The most common systemic adverse effect was fatigue. In the first dose, it was 30.3% for Sinopharm, 67.4% for AZD1222, 47.6% for Sputnik V, and 17.1% for Barekat. These rates were reduced to 24.6%, 37.1%, 36.5%, and 19.5%, in the second dose of vaccines. AZD1222 had the highest local and systemic adverse effects rates. The odds ratio of local adverse effects of the AZD1222 vaccine compared to the Sinopharm vaccine were 8.73 (95% CI 6.93–10.99) in the first dose and 4.14 (95% CI 3.32–5.17) in the second dose. Barekat and Sinopharm had the lowest frequency of local and systemic adverse effects. Compared to Sinopharm, systemic adverse effects were lower after the first dose of Barekat (OR = 0.56; 95% CI 0.46–0.67). Reactogenicity events were higher in women and younger people. Prior COVID-19 infection increased the odds of adverse effects only after the first dose of vaccines.

**Conclusions:**

Pain and fatigue were the most common reactogenicities of COVID-19 vaccination. Reactogenicities were less common after the second dose of the vaccines. The adverse effects of AZD1222 were greater than those of other vaccines.

## Background

The COVID-19 pandemic has caused significant mortality and morbidity worldwide, and until now, vaccination has been the most effective and promising strategy to control the spread of this disease [1, 2]. More than 250 vaccine production projects for COVID-19 have been launched worldwide since 2020 [3]. According to a recent World Health Organization (WHO) report, 176 vaccines are in clinical, and 199 vaccines are in the preclinical development phases. However, at least 27 vaccines have been clinically used or approved against SARS-CoV-2 [4] and as of 12 January 2022, nine vaccines have been authorized for emergency use by WHO [5].

The most common adverse effects of the vaccines include pain, swelling, redness at the injection site and fever, chills, headache, myalgia, fatigue, nausea, and joint pain as systemic adverse effects [6]. These reactogenicity events usually last 12 h to less than 7 days; in rare cases, they continue up to a month after vaccination [7, 8]. The local and systemic adverse effects are common but usually mild and self-limiting. Most of these reactions should resolve within a few days [9, 10]. However, they may be dangerous and cause fear in some cases. Besides, concerns about the adverse effects of COVID-19 vaccines may influence people’s decision to accept or reject the vaccine [11, 12].

COVID-19 vaccination was performed based on age groups from older to younger and prioritized high-risk groups. Many countries have commenced their vaccination program, prioritizing those most at risk due to the limited number of available vaccines [7]. In Iran, COVID-19 vaccination was started for high-risk groups, with Sinopharm, Sputnik V, AZD1222, and COVIran Barekat vaccines in high-risk groups and rolled out to other population groups.

It is essential to determine the adverse effects of new COVID-19 vaccines. The WHO has developed guidelines [13] for safety signal detection after vaccination and recommended it in a different setting. This study was performed based on this protocol and aimed to investigate the local and systemic adverse effects in an Iranian group of vaccinated individuals.

## Methods

### Study design and participant

This prospective observational study evaluated the reactogenicity adverse effects of COVID-19 vaccines, including Sinopharm (inactivated vaccine), Sputnik V (a human adenovirus vector-based vaccine), AZD1222 (a chimpanzee adenovirus vector-based vaccine), and COVIran Barekat (Inactivated vaccine), based on WHO protocol [13]. This study was performed in seven cities in Iran (Shahroud, Rasht, Zahedan, Sanandaj, Birjand, Kerman, and Mashhad) and its protocol has been published previously [14]. The study population included all eligible individuals who received one of the different types of COVID-19 vaccine according to the Iranian guidelines for COVID-19 vaccination. Signing the written informed consent by people vaccinated with the first dose of COVID-19 vaccines at one of the vaccination centers participating in the study was considered as inclusion criteria. Exclusion criteria were included: individuals who were already vaccinated with any COVID-19 vaccines before study enrolment, and unable to comply with study procedures. Participants had the right to withdraw from the study for any reason at any time.

The necessary information, including contact information, demographic characteristics, and history of underlying diseases (diabetes, hypertension, immunodeficiency, cancer, chronic heart disease, and respiratory, renal, hepatic, neurological, and psychiatric diseases) were collected during enrolment. Also, all the details of the injected vaccine, including the vaccine brand, vaccination date, and the vaccine’s batch number, were recorded in the designed registration system. Weight and height were also self-reported, and obesity was defined as a Body Mass Index (BMI) equal to or more than 30 kg/m^2^.

### Data collection

This study used telephone calls and electronic methods (mobile application and web pages) to collect data for at least 1000 participants of each vaccine. The local and systemic reactions after vaccination were recorded on days 1 to 7 after each dose of the vaccine. A reminder SMS was sent if the participants did not report the adverse effect data to the application by 16:00. If the data were not entered after the SMS, the trained experts actively followed and recorded the occurrence of adverse effects using telephone calls. For participants who were reluctant to use the web application, all data were collected by daily phone calls. Participants could also enter free textual reports about their post-vaccination experience and adverse events. In order to minimize loss to follow-up rate, the participants were contacted by phone up to twice a day. If they could not be reached, their next kin was followed up, and finally, if none of these worked, the call of that day was recorded as missed. A participant was considered lost to follow up after two unsuccessful attempts to contact them by phone, followed by one unsuccessful attempt to contact their next of kin.

### Outcomes

The main objective of this study was to estimate the reactogenicity within 7 days after each COVID-19 vaccine dose, and the primary outcome was the proportion of individuals who reported local or systemic adverse effects within 7 days of the first and second vaccine doses. The local and systemic reactogenicities included pain at the injection site, redness, swelling, induration, warmness, itching, fever, nausea, malaise, chills, headache, joint pain, myalgia, and fatigue. The severity of reactogenicities was also assessed for every reaction by asking about the extent to which adverse effects interfere with the participant’s daily activities.

### Statistical analysis

The proportion of systemic and local adverse effects within 7 days of vaccination was calculated and reported with 95% confidence intervals. Observed-to-expected analyses were performed for systemic reactogenicities using the collected data for the 3 days before vaccination. The duration (in days) was calculated for each type of event and their mean and standard deviation were reported. Separate logistic regression models were conducted for each vaccine dose to calculate and compare the odds ratio (OR) of local and systemic adverse effects while adjusting for age, sex, BMI, comorbidities and prior COVID-19 disease. The significance level was considered ≤ 0.05.

## Results

Out of 4639 people who received the first dose of vaccines from April 7, 2021, to January 11, 2022, 2908 (62.7%) received the second dose. The participants completed follow up in 7 days after vaccination with each dose of vaccines. The mean age of those who received the first dose was 46.7 (Standard Deviation [SD]: 18.5) years. The age and sex distribution of participants is provided in Table 1. Participants in the Sinopharm and Barekat groups had higher mean ages than other vaccine groups. The mean BMI of participants was 25.5 (SD: 4.3), which was higher in those receiving the Barekat [26.6 (SD: 4.2)] compared to participants receiving other vaccines (Table 1).

**Table 1:** The age and sex distribution of participants who had completed follow ups, and proportion with 95% confidence intervals (in parentheses) of at least one local and systemic adverse effects after vaccination by vaccine brands and doses

Adverse effects	Sputnik V		Sinopharm		AZD1222		COVIran Barekat	
	Dose 1	Dose 2	Dose 1	Dose 2	Dose 1	Dose 2	Dose 1	Dose 2
Number of participants	1253	823	1429	880	1010	840	950	365
Mean (SD) age (in year)	37.6 (10.7)	37.3 (10.9)	55.5 (22.8)	55.4 (24.0)	38.2 (15.1)	37.3 (13.7)	54.3 (12.1)	52.3 (14.2)
Sex [number (%)]								
Male	651 (52.0)	386 (46.9)	620 (43.4)	360 (41.0)	399 (39.5)	339 (40.4)	622 (65.5)	225 (61.6)
Female	602 (48.0)	437 (53.1)	809 (56.6)	520 (59.0)	611 (60.5)	501 (59.6)	328 (34.5)	140(38.4)
Body Mass Index	25.4 (4.0)	25.2 (4.0)	25.3 (4.6)	25.1 (4.4)	24.7 (4.0)	24.8 (4.0)	26.6 (4.2)	26.8 (4.3)
At least one solicited local adverse effect								
Total	78.5 (76.2–80.8)	64.8 (61.5–68.0)	38.6 (36.1–41.1)	29.1 (26.1–32.1)	87.4 (85.4–89.5)	68.3 (65.2–71.5)	33.4 (30.4–36.4)	50.9 (45.8–56.1)
Male	69.0 (65.4–72.5)	57.3 (52.3–62.2)	27.6 (24.1–31.2)	18.3 (14.3–22.3)	78.2 (74.1–82.3)	60.8 (55.6–66.0)	25.6 (22.1–29.0)	40.4 (34.0–46.9)
Female	88.8 (86.3–91.4)	71.4 (67.2–75.6)	47.0 (43.5–50.4)	36.5 (32.4–40.6)	93.5 (91.5–95.4)	73.5 (69.6–77.3)	48.2 (42.8–53.6)	67.9 (60.1–75.6)
At least one solicited systemic adverse effect								
Total	73.8 (71.4–76.3)	64.2 (60.9–67.4)	48.5 (45.9–51.1)	37.5 (34.3–40.7)	88.5 (86.5–90.5)	59.8 (56.4–63.0)	31.2 (28.3–34.2)	35.9 (31.0–40.8)
Male	67.1 (63.5–70.7)	59.3 (54.4–64.2)	38.5 (34.7–42.4)	28.6 (23.9– 33.3)	85.5 (82.0–88.9)	51.6 (46.3–57.0)	28.0 (24.4–31.5)	30.7 (24.6–36.7)
Female	81.1 (77.9–84.2)	68.4 (64.1–72.8)	56.1 (52.7–59.5)	43.7 (39.4–47.9)	90.5 (88.2–92.8)	65.3 (61.1–69.4)	37.5 (32.3–42.7)	44.3 (36.0–52.5)

Considering that the enrolment was started with high-risk groups and those with a history of underlying diseases, the prevalence of underlying diseases was high in participants with 62.6% (95% CI 61.2–64.0) having a history of underlying diseases.

**Fig. 1:**
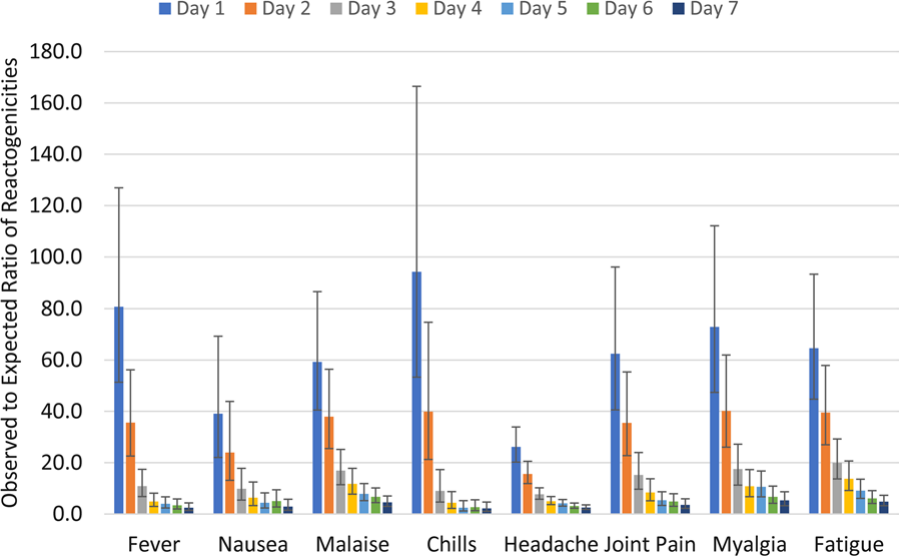
The observed to expected ratio of systemic adverse effects in 7 days after COVID-19 vaccination

Among first-dose recipients, 58.9% (95% CI 57.5–60.3) had one or more local adverse effects, and 60.5% (95% CI 59.1–61.9) had one or more systemic adverse effects. Among second-dose recipients, 53.1% (95% CI 51.2–55.0) had one or more local adverse effects, and 50.8% (95% CI 48.8–52.7) had one or more systemic adverse effects. The frequency of one or more local and systemic adverse effects was also highest in the first dose of the AZD1222 vaccine. Except for local adverse effects in Barekat recipients, the frequency of local and systemic adverse effects was lower after the second dose of vaccines compared to the first dose (Table 1). The observed systemic adverse effects were significantly higher than the expected rates. As depicted in Fig. 1, even on the 7th day after vaccination, the ratio of observed to expected systemic reactogenicities is high (nearly four), and its lower bounds are higher than one.

**Fig. 2:**
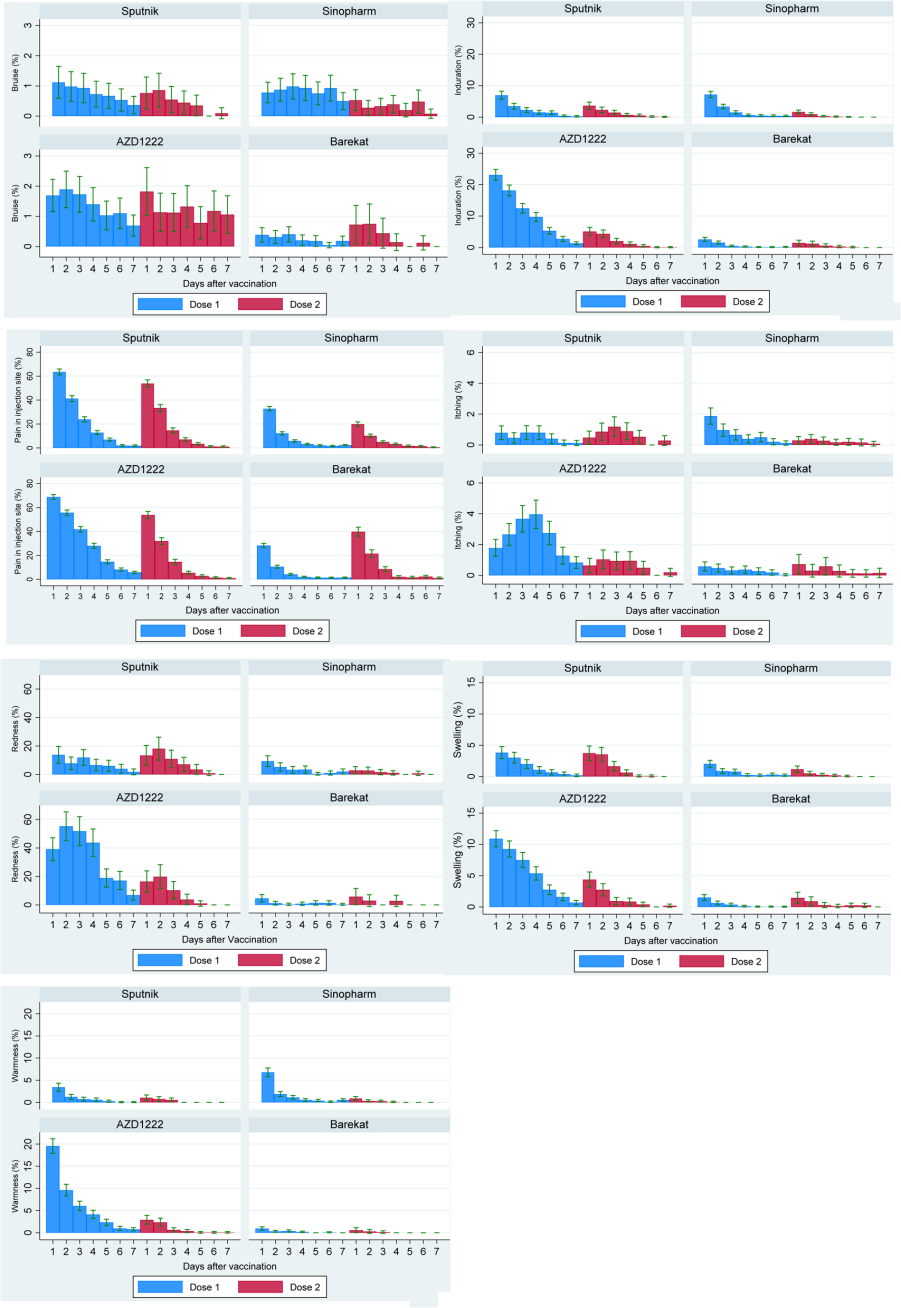
The frequency of local adverse effects in 7 days after COVID-19 vaccination by vaccine doses and vaccine brands

**Fig. 3:**
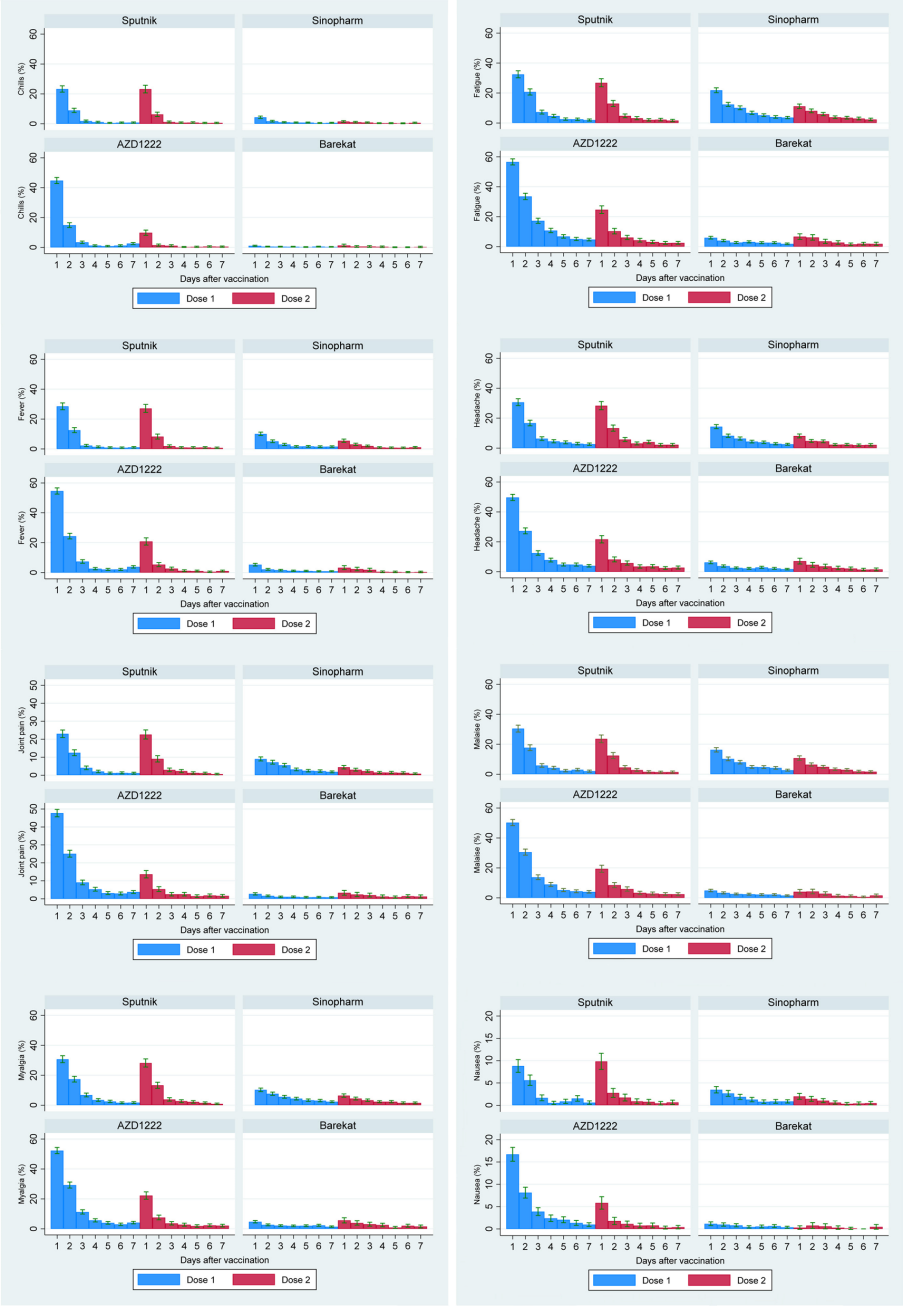
The frequency of systemic adverse effects in 7 days after COVID-19 vaccination by vaccine doses and vaccine brands

Figures 2 and 3 present the local and systemic adverse effects in different vaccine brands in 7 days after the first and second doses of vaccines. The most common local adverse effect reported in all vaccines was pain at the injection site, and the most common systemic adverse effect in all vaccines was fatigue. Most adverse effects had lower frequency after the second dose of vaccines. The systemic adverse effects were higher in each dose in the first 24 h after injection (Fig. 3). Except for redness, itching, and bruising, a similar pattern was also present for local adverse effects (Fig. 2). Compared to Sinopharm and Barekat, AZD1222 and Sputnik V had a higher frequency of local and systemic adverse effects (Table 2, Figs. 2 and 3).

**Table 2:** The frequency of local and systemic reactogenicity events in the 1–7 days after vaccination by vaccine doses and vaccine brands

Adverse effects	Sputnik V [% (95% CI)]		Sinopharm [% (95% CI)]		AZD1222 [% (95% CI)]		COVIran Barekat [% (95% CI)]	
	Dose 1	Dose 2	Dose 1	Dose 2	Dose 1	Dose 2	Dose 1	Dose 2
Pain	77.6 (75.2–79.9)	63.9 (60.5–67.2)	35.5 (33.0–38.0)	27.3 (24.4–30.4)	86.0 (83.7–88.1)	66.5 (63.2–69.7)	30.9 (28.0–34.0	49.0 (43.8–54.3)
Redness	2.3 (1.5–3.2)	3.8 (2.5–5.3)	0.6 (0.2–1.1)	0.6 (0.1–1.32)	9.9 (8.1–11.8)	4.0 (2.8–5.6)	0.3 (0.1–0.9)	1.1 (0.3–2.7)
Swelling	7.5 (6.1–9.1)	7.2 (5.5–9.1)	2.3 (1.6–3.2)	1.7 (0.9–2.8)	20.1 (17.6–22.7)	7.4 (5.7–9.3)	2.3 (1.4–3.4)	3.3 (1.7–5.6)
Induration	10.6 (8.9–12.4)	6.9 (5.2–8.8)	5.0 (3.9–6.2)	1.5 (0.7–2.5)	32.8 (29.8–35.7)	9.5 (7.6–1.1)	4.3 (3.1–5.8)	3.0 (1.5–5.3)
Bruise	2.7 (1.8–3.7)	2.2 (1.3–3.4)	1.5 (0.9–2.3)	1.6 (0.8–2.6)	4.3 (3.1–5.6)	3.9 (2.7–5.4)	1.2 (0.5–2.0)	1.9 (0.7–3.9)
Warmness	3.8 (2.8–5.0)	2.7 (1.6–4.0)	3.7 (2.7–4.8)	0.9 (0.3–1.7)	22.7 (20.0–25.3)	6.0 (4.4–7.7)	1.9 (1.1–2.9)	0.8 (0.1–2.3)
Itching	2.2 (1.4–3.1)	2.8 (1.7–4.1)	1.6 (1.0–2.4)	0.9 (0.3–1.7)	9.6 (7.8–11.5)	3.2 (2.1–4.6)	1.7 (0.9–2.7)	1.4 (0.4–3.1)
Fever	35.1 (32.4–37.8)	31.1 (27.9–34.3)	14.4 (12.6–16.3)	9.7 (7.7–11.8)	63.3 (60.2–66.2)	26.4 (23.4–29.5)	8.4 (6.7–10.3)	7.9 (5.3–11.2)
Nausea	13.3 (11.4–15.3)	12.5 (10.3–14.9)	7.8 (6.4–9.2)	4.8 (3.4–6.4)	26.0 (23.3–28.8)	9.0 (7.1–11.1)	2.8 (1.8–4.1)	1.9 (0.7–3.9)
Malaise	43.6 (40.8–46.3)	33.7 (30.4–37.0)	25.9 (23.6–28.2)	22.6 (19.8–25.5)	61.7 (58.6–64.6)	31.7 (28.5–34.9)	12.8 (10.7–15.1)	12.6 (9.3–16.4)
Chills	29.1 (26.5–31.6)	26.1 (23.1–29.2)	6.0 (4.8–7.3)	3.2 (2.1–4.5)	51.7 (48.6–54.8)	12.4 (10.2–14.7)	1.6 (0.8–2.5)	3.3 (1.7–5.6)
Headache	40.2 (37.4–43.0)	35.4 (32.0–38.7)	20.6 (18.5–22.7)	15.2 (12.9–17.7)	62.4 (59.3–65.3)	32.4 (29.2–35.6)	12.7 (10.6–15.0)	13.4 (10.1–17.3)
Joint pain	32.1 (29.5–34.7)	29.4 (26.3–32.6)	15.4 (13.5–17.3)	11.1 (9.1–13.4)	58.9 (55.8–61.9)	22.6 (19.8–25.6)	7.6 (5.9–9.4)	9.0 (6.3–12.4)
Myalgia	41.2 (38.4–43.9)	35.5 (32.2–38.8)	18.2 (16.2–20.2)	14.0 (11.7–16.4)	63.7 (60.6–66.6)	30.9 (27.8–34.1)	9.5 (7.6–11.5)	13.4 (10.1–17.3)
Fatigue	47.6 (44.8–50.4)	36.5 (33.1–39.8)	30.3 (27.9–32.7)	24.6 (21.8–27.6)	67.4 (64.4–70.3)	37.1 (33.8–40.4)	17.1 (14.7–19.6)	19.5 (15.5–23.8)

The average days with at least one local adverse effect after receiving all vaccines’ first and second doses was 1.81 and 1.05 days, respectively. It was higher for AZD1222 than other vaccines. Moreover, the average number of days with pain in injection site in the first and second doses were higher than other adverse effects. The average days with at least one systematic adverse effect in individuals after receiving the first and second doses of all vaccines were 3.71 and 2.87 days, respectively. Again, it was higher for AZD1222 than other vaccines. Also, the average number of days with fatigue in the first, and fever in the second dose were higher than other adverse effects (Table 3).

**Table 3:** The mean and standard deviation (SD) of duration (in days) of adverse effects after vaccination by vaccine brands

Adverse effects	Sputnik V		Sinopharm		AZD1222		COVIran Barekat	
	Dose 1	Dose 2	Dose 1	Dose 2	Dose 1	Dose 2	Dose 1	Dose 2
	Mean (SD)	Mean (SD)	Mean (SD)	Mean (SD)	Mean (SD)	Mean (SD)	Mean (SD)	Mean (SD)
Local								
Any	1.93 (2.14)	1.45 (1.91)	0.71 (1.35)	0.49 (1.0)	4.13 (4.48)	1.62 (2.05)	0.60 (1.27)	0.94 (1.38)
Pain	1.51 (1.25)	1.10 (1.12)	0.51 (0.87)	0.39 (0.78)	2.28 (1.65)	1.14 (1.10)	0.45 (0.82)	0.76 (0.98)
Redness	0.03 (0.29)	0.05 (0.31)	0.01 (0.78)	0.01 (0.11)	0.21 (0.75)	0.05 (0.27)	0.01 (0.56)	0.01 (0.16)
Swelling	0.10 (0.46)	0.9 (0.39)	0.3 (0.25)	0.2 (0.19)	0.37 (0.92)	0.9 (0.36)	0.2 (0.18)	0.4 (0.30)
Induration	0.14 (0.50)	0.9 (0.40)	0.06 (0.31)	0.01 (0.13)	0.66 (1.20)	0.13 (0.49)	0.04 (0.24)	0.04 (0.24)
Bruise	0.04 (0.35)	0.02 (0.22)	0.03 (0.32)	0.02 (0.25)	0.08 (0.48)	0.07 (0.50)	0.02 (0.27)	0.03 (0.23)
Warmness	0.04 (0.26)	0.02 (0.18)	0.04 (0.24)	0.01 (0.12)	0.35 (0.82)	0.07 (0.32)	0.02 (0.18)	0.01 (0.15)
Itching	0.02 (0.19)	0.04 (0.31)	0.02 (0.21)	0.01 (0.11)	0.15 (0.55)	0.04 (0.29)	0.02 (0.23)	0.02 (0.33)
Systemic								
Any	4.01 (4.38)	3.25 (3.98)	2.17 (3.86)	1.53 (2.95)	7.40 (6.07)	2.93 (4.47)	1.01 (2.43)	1.20 (2.49)
Fever	0.44 (0.69)	0.36 (0.60)	0.19 (0.55)	0.12 (0.41)	0.93 (0.91)	0.34 (0.66)	0.10 (0.39)	0.13 (0.51)
Nausea	0.17 (0.51)	0.15 (0.45)	0.11 (0.50)	0.05 (0.27)	0.37 (0.78)	0.11 (0.41)	0.03 (0.21)	0.02 (0.16)
Malaise	0.63 (0.90)	0.46 (0.78)	0.41 (0.86)	0.33 (0.73)	1.08 (1.19)	0.48 (0.90)	0.19 (0.58)	0.18 (0.54)
Chills	0.35 (0.61)	0.30 (0.55)	0.06 (0.29)	0.03 (0.23)	0.66 (0.75)	0.14 (0.41)	0.02 (0.16)	0.03 (0.19)
Headache	0.64 (0.98)	0.59 (1.02)	0.32 (0.75)	0.21 (0.58)	1.07 (1.15)	0.48 (0.88)	0.17 (0.56)	0.19 (0.57)
Joint pain	0.42 (0.73)	0.37 (0.64)	0.23 (0.69)	0.15 (0.50)	0.96 (1.09)	0.32 (0.75)	0.09 (0.37)	0.13 (0.49)
Myalgia	0.61 (0.89)	0.49 (0.77)	0.30 (0.78)	0.21 (0.62)	1.07 (1.10)	0.46 (0.89)	0.13 (0.47)	0.20 (0.61)
Fatigue	0.69 (0.90)	0.51 (0.79)	0.50 (0.97)	0.38 (0.82)	1.24 (1.25)	0.57 (0.97)	0.25 (0.68)	0.30 (0.71)

Compared to Sinopharm, both local and systemic adverse effects of AZD122 and Sputnik vaccines were higher after the first or second doses. The systemic adverse effects of Barekat were lower than Sinopharm (OR = 0.56, 95% CI 0.46–0.67) while its local adverse effects were similar to Sinopharm (P value: 0.969) after the first dose of vaccines. In the second dose, while the systemic adverse effects of Barekat were similar to Sinopharm (P value = 0.443), its local adverse effects were higher than Sinopharm (OR = 2.98, 95% CI 2.29–3.87). There were no significant differences between the local and systemic adverse effects of AZD1222 and Sputnik vaccines for the second doses. Local and systemic adverse effects of Barekat were lower than AZD1222 and Sputnik in both doses of vaccines (Table 4). Except for systemic adverse effects after the second dose of vaccines, local and systemic adverse effects decreased with an increase in age. All local and systemic adverse effects were higher in female participants. Prior COVID-19 disease increased the odds of local and systemic adverse effects only after the first dose of vaccines. Among the comorbidities, allergy and hypertension increased the odds of local adverse effects after the first dose of vaccines. Allergy, cardiac diseases, and cancers increased the odds of systemic adverse effects after the first dose of vaccines. Cancers were the only comorbidity that increased the odds of systemic adverse effects after the second dose of vaccines (OR = 1.87, 95% CI 1.25–2.80), as shown in Table 4.

**Table 4:** The associated factors with at least one solicited local or systemic adverse effects (AE) in multiple logistic regression models

Independent variables	Local AE after 1st dose		Systemic AE after 1st dose		Local AE after 2nd dose		Systemic AE after 2nd dose	
	OR (95% CI)	P value	OR (95% CI)	P value	OR (95% CI)	P value	OR (95% CI)	P value
Age	0.97 (0.97–0.98)	< 0.001	0.98 (0.97–0.98)	< 0.001	0.98 (0.97–0.98)	< 0.001	0.99 (0.99–1.00)	0.298
Female sex	2.65 (2.29–3.07)	< 0.001	1.78 (1.55–2.04)	< 0.001	2.07 (1.76–2.43)	< 0.001	1.69 (1.45–1.98)	< 0.001
Prior COVID-19	1.32 (1.17–1.50)	< 0.001	1.20 (1.07–1.35)	0.001	1.07 (0.94–1.22)	0.291	1.08 (0.96–1.23)	0.187
Vaccine brands								
Sinopharm	Reference	–	Reference	–	Reference	–	Reference	–
Sputnik V	4.74 (3.90–5.76)	< 0.001	2.54 (2.11–3.06)	< 0.001	3.64 (2.91–4.55)	< 0.001	3.19 (2.55–3.99)	< 0.001
AZD1222	8.73 (6.93–10.99)	< 0.001	6.79 (5.39–8.57)	< 0.001	4.14 (3.32–5.17)	< 0.001	2.56 (2.06–3.18)	< 0.001
COVIran Barekat	1.00 (0.83–1.20)	0.969	0.56 (0.46–0.67)	< 0.001	2.98 (2.29–3.87)	< 0.001	1.10 (0.85–1.44)	0.443
Comorbidities								
Allergy	2.18 (1.16–4.08)	0.015	2.47 (1.34–4.56)	0.004	NR	–	NR	–
Hypertension	1.25 (1.02–1.55)	0.032	NR	–	NR	–	NR	–
Cardiac diseases	NR	–	1.55 (1.22–1.97)	< 0.001	NR	–	NR	–
Cancer	NR	–	1.73 (1.13–1.91)	0.006	NR	–	1.87 (1.25–2.80)	0.002

*NR* not retained in the multiple logistic regression models, *OR* odds ratio, *CI* confidence intervals

Multiple Logistic regression results for the odds of local and systemic side effects after the first dose of vaccines are shown in Table 5. All local and systemic adverse effects were higher in female participants and decreased with an increase in the age of participants. The odds of redness, induration, itching and swelling were higher in obese participants. Prior COVID-19 disease increased the odds of pain at injection site and systemic adverse effects except for nausea and fever. Headache was lower in participants with comorbidities (OR = 0.81, 95% CI 0.69–0.96).

**Table 5:** The association of age, sex, obesity, commodities, and prior COVID-19 infection with local and systemic reactogenicities following the first dose of all vaccines in multiple logistic regression

Adverse effects	Female sex		Age (year)		Commodities		Prior COVID-19 disease		Obesity	
	OR (95% CI)	P value	OR (95% CI)	P value	OR (95% CI)	P value	OR (95% CI)	P value	OR (95% CI)	P value
Local										
Pain	2.54 (2.22–2.91)	< 0.001	0.95 (0.95–0.96)	< 0.001	1.08 (0.92–1.27)	0.327	1.79 (1.50–2.12)	< 0.001	0.85 (0.70–1.03)	0.099
Redness	3.20 (2.13–4.81)	< 0.001	0.96 (0.95–0.97)	< 0.001	0.80 (0.52–1.22)	0.309	1.28 (0.87–1.88)	0.199	2.05 (1.30–3.22)	0.002
Swelling	2.37 (1.85–3.04)	< 0.001	0.97 (0.96–0.97)	< 0.001	1.07 (0.80–1.44)	0.613	1.29 (1.00–1.67)	0.046	1.51 (1.09–2.09)	0.012
Induration	2.28 (1.87–2.77)	< 0.001	0.96 (0.95–0.97)	< 0.001	0.81 (0.64–1.02)	0.077	1.17 (0.94–1.44)	0.146	1.30 (1.00–1.71)	0.048
Bruise	2.11 (1.38–3.22)	0.001	0.98 (0.97–0.99)	0.007	0.67 (0.42–1.08)	0.103	1.08 (0.68–1.72)	0.725	0.60 (0.30–1.22)	0.162
Warmness	2.29 (1.79–2.93)	< 0.001	0.96 (0.95–0.97)	< 0.001	1.18 (0.87–1.59)	0.269	1.02 (0.78–1.33)	0.872	1.16 (0.81–1.66)	0.398
Itching	2.28 (1.60–3.24)	< 0.001	0.97 (0.96–0.98)	< 0.001	1.03 (0.69–1.55)	0.866	1.17 (0.80–1.69)	0.404	1.62 (1.05–2.52)	0.029
Systemic										
Fever	1.57 (1.37–1.80)	< 0.001	0.96 (0.96–0.97)	< 0.001	1.04 (0.88–1.23)	0.610	1.15 (0.98–1.35)	0.084	1.01 (0.82–1.24)	0.877
Nausea	2.91 (2.37–3.57)	< 0.001	0.97 (0.96–0.97)	< 0.001	0.85 (0.67–1.07)	0.176	1.04 (0.83–1.30)	0.702	0.97 (0.72–1.29)	0.839
Malaise	1.83 (1.61–2.08)	< 0.001	0.97 (0.97–0.98)	< 0.001	0.99 (0.84–1.16)	0.933	1.22 (1.04–1.42)	0.010	0.94 (0.78–1.14)	0.579
Chills	1.61 (1.38–1.87)	< 0.001	0.96 (0.95–0.96)	< 0.001	1.14 (0.94–1.38)	0.170	1.24 (1.04–1.48)	0.014	1.12 (0.89–1.40)	0.325
Headache	1.85 (1.62–2.11)	< 0.001	0.96 (0.96–0.97)	< 0.001	0.81 (0.69–0.96)	0.016	1.23 (1.05–1.44)	0.008	0.95 (0.78–1.16)	0.641
Joint pain	1.87 (1.62–2.15)	< 0.001	0.97 (0.96–0.97)	< 0.001	1.04 (0.88–1.24)	0.602	1.25 (1.06–1.47)	0.007	0.84 (0.68–1.04)	0.115
Myalgia	1.68 (1.47–1.92)	< 0.001	0.96 (0.96–0.97)	< 0.001	0.94 (0.80–1.11)	0.506	1.23 (1.05–1.44)	0.008	0.92 (0.76–1.13)	0.466
Fatigue	1.65 (1.45–1.86)	< 0.001	0.97 (0.96–0.97)	< 0.001	0.88 (0.75–1.02)	0.11	1.18 (1.01–1.37)	0.033	0.97 (0.81–1.17)	0.790

*OR* odds ratio, *CI* confidence intervals

In another multiple logistic regression models the associated factors with local and systemic adverse effects after the second dose of vaccines were investigated and presented in Table 6. The results were almost similar to the above findings for the first dose. All local and systemic adverse effects were higher in female participants. Except for warmness, itching and swelling, other local and systemic adverse effects decreased with an increase in age. Comorbidities only increased the odds of pain at injection site (P value = 0.048) and were not associated with other local and systemic adverse effects. Obesity only increased the odds of redness after the second dose of vaccines. Prior COVID-19 disease increased the odds of pain at injection site and all systemic adverse effects except nausea.

**Table 6:** The association of age, sex, obesity, commodities, and prior COVID-19 infection with local and systemic reactogenicities following the second dose of all vaccines in multiple logistic regression

Adverse effects	Female sex		Age (year)		Commodities		Prior COVID-19 disease		Obesity	
	OR (95% CI)	P value	OR (95% CI)	P value	OR (95% CI)	P value	OR (95% CI)	P value	OR (95% CI)	P value
Local										
Pain	1.81 (1.55–2.12)	< 0.001	0.97 (0.96–0.99)	< 0.001	1.20 (1.00–1.45)	0.048	1.43 (1.18–1.73)	< 0.001	0.92 (0.73–1.17)	0.527
Redness	3.15 (1.77–5.61)	< 0.001	0.97 (0.95–0.99)	0.005	1.12 (0.62–2.04)	0.698	1.11 (0.65–1.90)	0.693	1.93 (1.02–3.63)	0.042
Swelling	2.79 (1.88–4.14)	< 0.001	0.98 (0.97–1.00)	0.069	1.33 (0.86–2.05)	0.195	1.07 (0.72–1.59)	0.704	1.28 (0.77–2.10)	0.331
Induration	2.20 (1.53–3.16)	< 0.001	0.98 (0.97–0.99)	0.003	1.14 (0.75–1.72)	0.526	0.85 (0.57–1.27)	0.433	1.48 (0.93–2.36)	0.093
Bruise	3.22 (1.78–5.82)	< 0.001	0.97 (0.96–0.99)	0.006	0.68 (0.39–1.18)	0.177	1.27 (0.75–2.16)	0.367	0.81 (0.36–1.82)	0.620
Warmness	3.22 (1.85–5.62)	< 0.00	0.98 (0.97–1.00)	0.101	1.06 (0.60–1.87)	0.818	0.76 (0.43–1.36)	0.365	1.14 (0.57–2.27)	0.706
Itching	2.58 (1.43–4.65)	0.002	0.98 (0.96–1.00)	0.094	1.00 (0.53–1.86)	0.994	1.32 (0.75–2.31)	0.322	1.26 (0.62–2.68)	0.494
Systemic										
Fever	1.56 (1.29–1.90)	< 0.001	0.97 (0.97–0.98)	< 0.001	1.05 (0.84–1.33)	0.629	1.32 (1.06–1.63)	0.010	1.19 (0.90–1.58)	0.206
Nausea	2.83 (2.04–3.91)	< 0.001	0.98 (0.97–0.99)	< 0.001	0.82 (0.59–1.16)	0.273	1.29 (0.94–1.77)	0.106	0.60 (0.35–1.00)	0.054
Malaise	1.74 (1.47–2.07)	< 0.001	0.99 (0.98–0.99)	0.008	0.90 (0.73–1.10)	0.322	1.39 (1.14–1.12)	0.001	0.86 (0.66–1.12)	0.273
Chills	1.45 (1.15–1.84)	0.002	0.97 (0.96–0.98)	< 0.001	1.22 (0.91–1.63)	0.180	1.38 (1.07–1.78)	0.011	1.26 (0.89–1.77)	0.181
Headache	1.90 (1.59–2.27)	< 0.001	0.98 (0.98–0.99)	< 0.001	1.13 (0.91–1.40)	0.244	1.35 (1.10–1.64)	0.003	0.95 (0.73–1.25)	0.756
Joint pain	1.54 (1.27–1.88)	< 0.001	0.98 (0.97–0.98)	< 0.001	0.91 (0.72–1.15)	0.474	1.37 (1.11–1.70)	0.004	0.89 (0.66–1.20)	0.454
Myalgia	1.57 (1.32–1.88)	< 0.001	0.98 (0.97–0.98)	< 0.001	0.92 (0.74–1.13)	0.445	1.30 (1.06–1.59)	0.010	0.95 (0.73–1.25)	0.742
Fatigue	1.62 (1.37–1.91)	< 0.001	0.99 (0.98–0.99)	0.001	0.88 (0.72–1.07)	0.226	1.25 (1.03–1.51)	0.020	1.03 (0.81–1.31)	0.785

*OR* odds ratio, *CI* confidence intervals

**Fig. 4:**
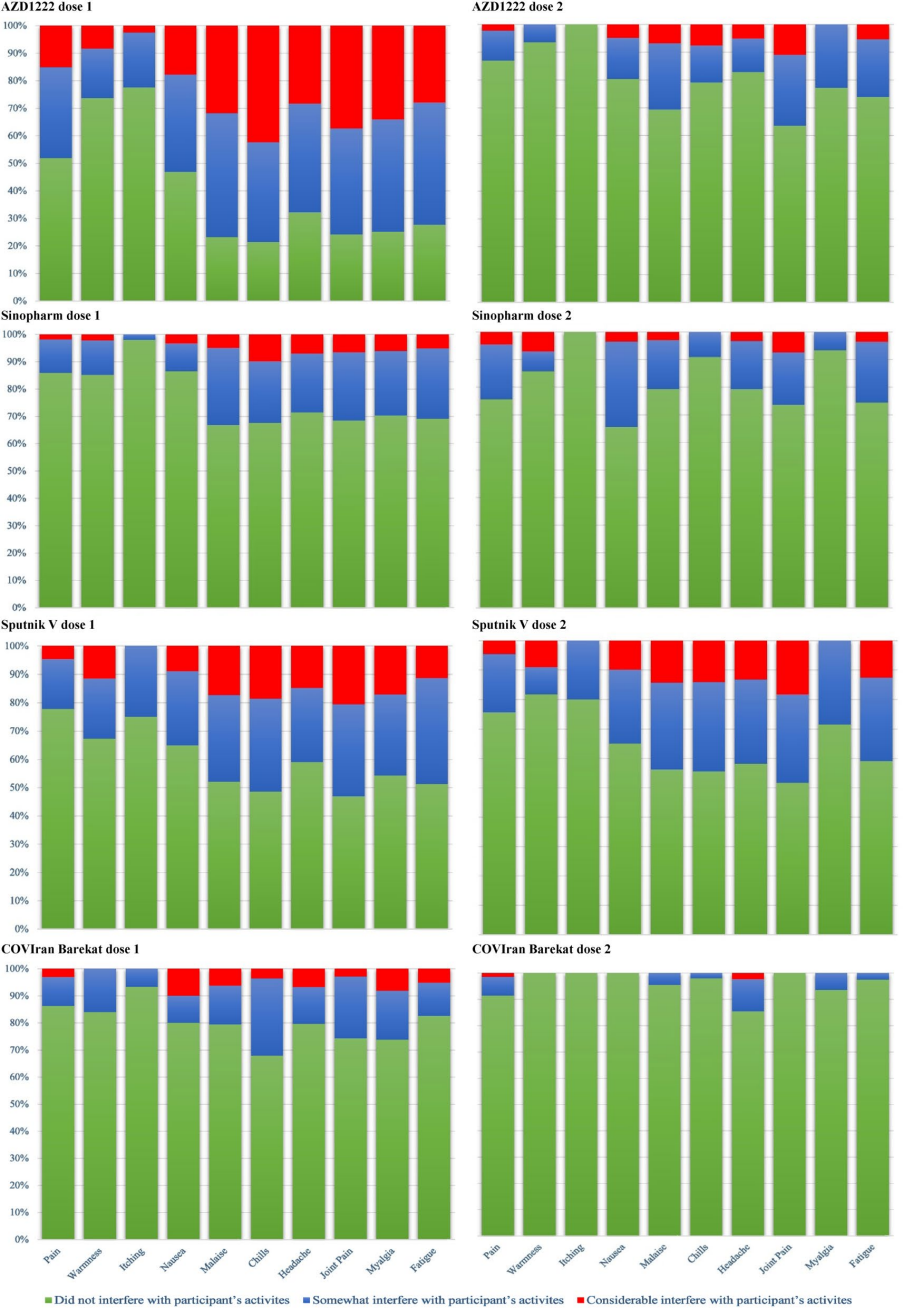
The severity of adverse effects in the first days after the first and second doses of COVID-19 vaccines

The findings indicate that the local and systemic adverse effects in all vaccines did not interfere with or even partially interfere with participants’ daily activities. Also, after receiving the second dose of vaccines, the interference with daily activities is less than the first dose. The severity of adverse effects in the Barekat vaccine was lower than the other three vaccines, and the malaise, chills, headache, and myalgia interfered more with people’s daily activities than other adverse effects. Besides, these side effects were reported more in the first dose of AZD1222 (Fig. 4).

## Discussion

In this study, performed in several cities in Iran, the local and systemic reactogenicities of the COVID-19 vaccines were investigated. AZD1222 and Sputnik had highest local and systemic adverse effects frequency, while most adverse effects were the lowest in Barekat recipients. Except for AZD1222, the incidence of local and systemic adverse effects was mild to moderate and did not interfere with the daily activities of most individuals. The adverse effects in the second dose were less than in the first. Similar to our findings other studies reported a higher rate of reactogenicity after the first dose of AstraZeneca [15, 16] and Sputnik V [17]. Adverse events after the first dose of Janssen vaccine were also higher than its second dose [16]. However, in some studies [8, 18–20], it has been shown that adverse effects in the second dose were more than in the first dose. This could be due to the nature of the vaccines used, the response of the individual's immune system, the study methods and location, and age and sex differences between studies. Considering our results and the findings of a systematic review and meta-analysis study [21], it can be confirmed that first dose of adenovirus vectored vaccines is more reactogenic than the second one. For the mRNA and protein subunit vaccines, the opposite is true. For the Sinopharm, we also find similar results to AZD1222 and Sputnik regarding comparing adverse effects in two doses of vaccines. This finding was similar to the results of another study in the UAE [22]. However, in another inactivated vaccine (Barekat), the frequency of at least one local adverse effect was higher after the second dose, and for the systemic adverse effects, the difference between the two doses was not significant. This pattern was similar to the results of another study on CoronaVac, which is an inactivated COVID-19 vaccine [23]. It seems that the pain in injection site, which was higher after the second dose of the Barekat vaccine, caused a higher frequency of at least one local adverse effect after the second dose of Barekat. Considering the limited evidence for reactogenicity events of the Barekat vaccine, more studies with a higher sample size are needed to justify the above findings. Another study in Turkey [24], showed a higher incidence of reactogenicities after the second dose of Covaxine (an inactivated vaccine similar to Barekat) and a lower incidence of reactogenicities after the second dose of Covishield.

After each dose, the most commonly reported reactions were pain at the injection site and fatigue, followed by malaise in all vaccines. Various studies [8, 17, 25–31] showed that pain at the injection site is the most common local reactogenicity reported. Also, studies conducted in the third phase of the clinical trials [32, 33] indicated that pain at the injection site was reported as the most common complication. Moreover, injection site pain has been commonly reported as a local reaction in other COVID-19 vaccines [34].

Adverse effects after the AZD1222 vaccination were higher than other vaccines. Other studies also showed similar findings [8, 30, 35]. It is believed that the high local and systemic adverse effects of AZD1222 might be because it is a non-reproducible adenovirus carrier vaccine and uses a protein similar to the protein produced by the SARS-CoV2 virus following a natural infection [36, 37]. Lower frequency of adverse effects after vaccination with Barekat and Sinopharm can be attributed to their nature, which are inactivated vaccines. Many other studies [21–24, 35] also reported a lower frequency of local and systemic adverse effects in recipients of inactivated vaccines. Differences in vaccine platforms and structures, immunogenicity, and mechanism of action are the main reasons for the discrepancy between the reactogenicities of COVID-19 vaccines.

This study found that pain at the injection site and fatigue were the most common local and systemic adverse effects of the Sinopharm vaccine, which was consistent with studies carried out in the Czech Republic [38], Iraq [35], China [39], and the United Arab Emirates [31]. The most common adverse effects of the Sputnik V vaccine were also pain at the injection site and fatigue. In a clinical trial conducted in Russia [7], this vaccine’s most common adverse effects were pain at the injection site, fever, and chills. Also, in a study conducted on health workers [17], it was shown that pain at the injection site and fatigue were the most common adverse effects of Sputnik V and these reactogenicities were significantly more common in women and young people. In the current study, similar to other vaccines, the most common adverse effects of Barekat were pain at the injection site and fatigue.

Several risk factors related to local and systemic adverse effects after vaccination were identified in the present study. These risk factors include younger age, female sex, and BMI greater than 30 kg/m^2^. These findings are similar to the findings of studies carried out in the Czech Republic [36], Netherlands [40], Iraq [35], the United Kingdom [8], Saudi Arabia [27], Jordan [28, 41, 42], India [29] as well as the findings of the third phase of several clinical trials [32, 33, 43]. However, a study in Saudi Arabia [25] showed that the reactogenicities were higher in men than women, possibly due to the high proportion of men participating in that study [43].

The female gender was considered a significant risk factor for adverse effects following vaccination. Women generally have more robust immune responses than men [43]. Hence, they are more likely to have frequent and severe adverse effects. This difference may be related to genetic or hormonal differences between women and men [44].

In this study, allergy, hypertension, cardiac diseases and cancer were the underlying diseases that increased the odds of adverse effects. Other studies in Iraq [35] and the Netherlands [40] showed that asthma, hypertension, diabetes, and respiratory diseases are significant risk factors for post-vaccination adverse effects. Similarly, food and/or drug allergies and chronic diseases were associated with a higher frequency of post-vaccination side effects [45–47]. On the other hand, in a study done in France [26], no association was observed between disease history and vaccines’ reactogenicity. Although most studies showed a positive association between the presence of chronic diseases and reactogenicities, the underlying mechanisms are unclear. Interaction of vaccines with medications used, different immunological responses, better reporting and perceptions of adverse effects, and lower tolerance to adverse effects (in the case of cancers) are among the proposed mechanisms which should be investigated exclusively. The differences in the age groups, vaccine brands, prevalence of comorbidities, and sample size may be the reasons for the difference in studies’ results.

Our results showed that prior COVID-19 infection increased the odds of local and systemic adverse effects only after the first dose of vaccines. A study in Mexico on people who received the BNT162b2 vaccine [48], and another on BNT162b2, mRNA-1273 and Ad26.COV2.S vaccines [20] also reported similar findings. Higher T-cell and antibody responses in participants with a history of COVID-19 infection may be the reason for this finding. It has been shown that T-cell responses and anti-spike antibodies were higher after the first dose of the BNT162b2 vaccine in people with prior COVID-19 infection compared to infection-naive people. These responses were similar after two doses of the vaccine in infection-naive people and people with prior COVID-19 infection [49]. In fact, the first vaccine dose boosts the immune responses in people with prior COVID-19 infection, while the second vaccine dose results in little increase in immune responses [50]. Finally, other studies reported a higher frequency of adverse effects in participants with prior COVID-19 infection [8, 35, 40, 51, 52].

In the current study, most reported local and systemic adverse effects were mild to moderate in severity. In a clinical trial [32] on the AZD1222 vaccine and another study in Saudi Arabia [25], it was observed that the severity of adverse effects was mild to moderate. Also, in other studies on the Sinopharm vaccine [28, 29, 31, 41], the adverse effects have been mild. The severity of local and systemic adverse effects is influenced by the nature of the vaccines [28], the number of doses received, and the age and gender of participants.

The present study has various strengths, including using a standard protocol provided by WHO, active daily contact and direct monitoring of the study’s implementation, comparing four different vaccines, and using online methods and telephone calls to report adverse effects. However, the sample size for the second dose of Barekat did not reach 1000 participants, which might be a limitation of the current study. As another limitation, the participants’ weight and height did not measure and were based on self-reporting.

## Conclusions

In this study, adverse effects after vaccination (both systemic and local) often had the highest incidence in 1 to 2 days after vaccination and reached their lowest level at the end of the first week. Besides, pain at the injection site and fatigue were the most common reactogenicities of COVID-19 vaccination. However, most local and systemic adverse effects were not severe and did not interfere with people’s daily activities. AZD1222 and Sputnik had higher adverse effects frequencies than Sinopharm and Barekat vaccines. Furthermore, younger age, female gender, some comorbidities, and prior COVID-19 infection were associated with higher reactogenicities.

## Data Availability

All data of this study can be provided at the request of the corresponding author (Prof. Mohammad Hassan Emamian, via emamian@shmu.ac.ir). All researchers around the world can send their proposed titles. After screening in a scientific committee, the new titles will be approved and the required data will be available for researchers. The new articles and reports then will be prepared by collaboration with researchers of this study.

## Abbreviations

CI: Confidence intervals
WHO: World Health Organization
CDC: Centers for Disease Control and Prevention
BMI: Body Mass Index
OR: Odds ratio
SD: Standard deviation

## References

[1] Polack FP, Thomas SJ, Kitchin N, Absalon J, Gurtman A, Lockhart S (2020). Safety and efficacy of the BNT162b2 mRNA Covid-19 vaccine. N Engl J Med.

[2] Stringhini S, Wisniak A, Piumatti G, Azman AS, Lauer SA, Baysson H (2020). Seroprevalence of anti-SARS-CoV-2 IgG antibodies in Geneva, Switzerland (SEROCoV-POP): a population-based study. Lancet.

[3] Forni G, Mantovani A (2021). COVID-19 vaccines: where we stand and challenges ahead. Cell Death Differ.

[4] World Health Organization. Draft landscape of COVID-19 candidate vaccines 2023. https://www.who.int/publications/m/item/draft-landscape-of-covid-19-candidate-vaccines. Accessed 8 Jan 2023.

[5] World Health Organization. Coronavirus disease (COVID-19): vaccines. 2022. https://www.who.int/news-room/questions-and-answers/item/coronavirus-disease-(covid-19)-vaccines. Accessed 8 Jan 2023.

[6] Anderson EJ, Rouphael NG, Widge AT, Jackson LA, Roberts PC, Makhene M (2020). Safety and immunogenicity of SARS-CoV-2 mRNA-1273 vaccine in older adults. N Engl J Med.

[7] Logunov D, Dolzhikova I, Shcheblyakov D, Tukhvatulin A, Zubkova O, Dzharullaeva A (2021). Safety and efficacy of an rAd26 and rAd5 vector-based heterologous prime-boost COVID-19 vaccine: an interim analysis of a randomised controlled phase 3 trial in Russia. Lancet.

[8] Menni C, Klaser K, May A, Polidori L, Capdevila J, Louca P (2021). Vaccine side-effects and SARS-CoV-2 infection after vaccination in users of the COVID symptom study app in the UK: a prospective observational study. Lancet Infect Dis.

[9] Centre for Disease Control Prevention (CDC). Possible Side Effects After Getting a COVID-19. Updated Sept. 14, 2022. https://www.cdc.gov/coronavirus/2019-ncov/vaccines/expect/after.html. Accessed 23 Feb 2023.

[10] Gee J, Marquez P, Su J, Calvert GM, Liu R, Myers T (2021). First month of COVID-19 vaccine safety monitoring—United States, December 14, 2020–January 13, 2021. Morb Mortal Wkly Rep.

[11] Mulligan MJ, Lyke KE, Kitchin N, Absalon J, Gurtman A, Lockhart S (2020). Phase I/II study of COVID-19 RNA vaccine BNT162b1 in adults. Nature.

[12] Walsh EE, Frenck RW, Falsey AR, Kitchin N, Absalon J, Gurtman A (2020). Safety and immunogenicity of two RNA-based Covid-19 vaccine candidates. N Engl J Med.

[13] World Health Organization. Protocol template to be used as template for observational study protocols: sentinel surveillance of adverse events of special interest (AESIs) after vaccination with COVID-19 vaccines, 2021. https://apps.who.int/iris/bitstream/handle/10665/342194/9789240029507-eng.pdf?sequence=1.

[14] Aliyari R, Mahdavi S, Enayatrad M, Sahab-Negah S, Nili S, Fereidooni M (2022). Study protocol: cohort event monitoring for safety signal detection after vaccination with COVID-19 vaccines in Iran. BMC Public Health.

[15] Nachtigall I, Bonsignore M, Hohenstein S, Bollmann A, Günther R, Kodde C (2022). Effect of gender, age and vaccine on reactogenicity and incapacity to work after COVID-19 vaccination: a survey among health care workers. BMC Infect Dis.

[16] Kant A, Jansen J, van Balveren L, van Hunsel F (2022). Description of frequencies of reported adverse events following immunization among four different COVID-19 vaccine brands. Drug Saf.

[17] Babamahmoodi F, Saeedi M, Alizadeh-Navaei R, Hedayatizadeh-Omran A, Mousavi SA, Ovaise G (2021). Side effects and Immunogenicity following administration of the Sputnik V COVID-19 vaccine in health care workers in Iran. Sci Rep.

[18] Andrzejczak-Grządko S, Czudy Z, Donderska M (2021). Side effects after COVID-19 vaccinations among residents of Poland. Eur Rev Med Pharmacol Sci.

[19] Saita M, Yan Y, Ito K, Sasano H, Seyama K, Naito T (2022). Reactogenicity following two doses of the BNT162b2 mRNA COVID-19 vaccine: real-world evidence from healthcare workers in Japan. J Infect Chemother.

[20] Scher AI, Berjohn CM, Byrne C, Colombo RE, Colomba CJ, Edwards MS (2022). An analysis of SARS-CoV-2 vaccine reactogenicity: variation by type, dose, and history, severity, and recency of prior SARS-CoV-2 infection. Open Forum Infect Dis.

[21] Sutton N, San Francisco Ramos A, Beales E, Smith D, Ikram S, Galiza E (2022). Comparing reactogenicity of COVID-19 vaccines: a systematic review and meta-analysis. Expert Rev Vaccines.

[22] Ganesan S, Al Ketbi LMB, Al Kaabi N, Al Mansoori M, Al Maskari NN, Al Shamsi MS (2022). Vaccine side effects following COVID-19 vaccination among the residents of the UAE. An observational study. Front Public Health.

[23] Lai FTT, Leung MTY, Chan EWW, Huang L, Lau LKW, Peng K (2022). Self-reported reactogenicity of CoronaVac (Sinovac) compared with Comirnaty (Pfizer-BioNTech): a prospective cohort study with intensive monitoring. Vaccine.

[24] Riad A, Sağıroğlu D, Üstün B, Pokorná A, Klugarová J, Attia S (2021). Prevalence and risk factors of CoronaVac side effects: an independent cross-sectional study among healthcare workers in Turkey. J Clin Med.

[25] Al Bahrani S, Albarrak A, Alghamdi OA, Alghamdi MA, Hakami FH, Al Abaadi AK (2021). Safety and reactogenicity of the ChAdOx1 (AZD1222) COVID-19 vaccine in Saudi Arabia. Int J Infect Dis.

[26] Chouchana L, Canouï E, Batista R, Contejean A, Cariou A, Treluyer JM (2021). Balancing the reactogenicity of the ChAdOx1 nCov-19 vaccine against SARS-CoV-2 and the urgent need of a large immunization in healthcare workers. Therapie.

[27] El-Shitany NA, Harakeh S, Badr-Eldin SM, Bagher AM, Eid B, Almukadi H (2021). Minor to moderate side effects of Pfizer-BioNTech COVID-19 vaccine among Saudi residents: a retrospective cross-sectional study. Int J Gen Med.

[28] Hatmal MM, Al-Hatamleh MAI, Olaimat AN, Hatmal M, Alhaj-Qasem DM, Olaimat TM (2021). Side effects and perceptions following COVID-19 vaccination in Jordan: a randomized, cross-sectional study implementing machine learning for predicting severity of side effects. Vaccines (Basel).

[29] Jayadevan R, Shenoy R, Anithadevi T (2021). Survey of symptoms following COVID-19 vaccination in India. medRxiv.

[30] Mahallawi WH, Mumena WA (2021). Reactogenicity and immunogenicity of the Pfizer and AstraZeneca COVID-19 vaccines. Front Immunol.

[31] Saeed BQ, Al-Shahrabi R, Alhaj SS, Alkokhardi ZM, Adrees AO (2021). Side effects and perceptions following Sinopharm COVID-19 vaccination. Int J Infect Dis.

[32] Ramasamy MN, Minassian AM, Ewer KJ, Flaxman AL, Folegatti PM, Owens DR (2021). Safety and immunogenicity of ChAdOx1 nCoV-19 vaccine administered in a prime-boost regimen in young and old adults (COV002): a single-blind, randomised, controlled, phase 2/3 trial. Lancet.

[33] Sadoff J, Le Gars M, Shukarev G, Heerwegh D, Truyers C, de Groot AM (2021). Interim results of a phase 1–2a trial of Ad26. COV2. S Covid-19 vaccine. N Engl J Med.

[34] McDonald I, Murray SM, Reynolds CJ, Altmann DM, Boyton RJ (2021). Comparative systematic review and meta-analysis of reactogenicity, immunogenicity and efficacy of vaccines against SARS-CoV-2. NPJ Vaccines.

[35] Almufty HB, Mohammed SA, Abdullah AM, Merza MA (2021). Potential adverse effects of COVID19 vaccines among Iraqi population; a comparison between the three available vaccines in Iraq; a retrospective cross-sectional study. Diabetes Metab Syndr.

[36] Jahan N, Rahman FI, Saha P, Ether SA, Roknuzzaman A, Sarker R (2021). Side effects following administration of the first dose of Oxford-AstraZeneca’s Covishield vaccine in Bangladesh: a cross-sectional study. Infect Dis Rep.

[37] Rice SM, Ferree SD, Mesinkovska NA, Kourosh AS (2021). The art of prevention: COVID-19 vaccine preparedness for the dermatologist. Int J Womens Dermatol.

[38] Riad A, Pokorná A, Attia S, Klugarová J, Koščík M, Klugar M (2021). Prevalence of COVID-19 vaccine side effects among healthcare workers in the Czech Republic. J Clin Med.

[39] Zhu FC, Li YH, Guan XH, Hou LH, Wang WJ, Li JX (2020). Safety, tolerability, and immunogenicity of a recombinant adenovirus type-5 vectored COVID-19 vaccine: a dose-escalation, open-label, non-randomised, first-in-human trial. Lancet.

[40] Rolfes L, Härmark L, Kant A, van Balveren L, Hilgersom W, van Hunsel F (2022). COVID-19 vaccine reactogenicity—a cohort event monitoring study in the Netherlands using patient reported outcomes. Vaccine.

[41] Al Khames Aga QA, Alkhaffaf WH, Hatem TH, Nassir KF, Batineh Y, Dahham AT (2021). Safety of COVID-19 vaccines. J Med Virol.

[42] Omeish H, Najadat A, Al-Azzam S, Tarabin N, Abu Hameed A, Al-Gallab N (2021). Reported COVID-19 vaccines side effects among Jordanian population: a cross sectional study. Hum Vaccines Immunother.

[43] Voysey M, Clemens S, Madhi S (2021). Safety and efficacy of the ChAdOx1 nCoV-19 vaccine (AZD1222) against SARS-CoV-2: an interim analysis of four randomised controlled trials in Brazil, South Africa, and the UK. Lancet.

[44] Hervé C, Laupèze B, Del Giudice G, Didierlaurent AM, Tavares Da Silva F (2019). The how’s and what’s of vaccine reactogenicity. npj Vaccines.

[45] Hatmal MM, Al-Hatamleh MAI, Olaimat AN, Mohamud R, Fawaz M, Kateeb ET (2022). Reported adverse effects and attitudes among Arab populations following COVID-19 vaccination: a large-scale multinational study implementing machine learning tools in predicting post-vaccination adverse effects based on predisposing factors. Vaccines (Basel).

[46] Alghamdi AN, Alotaibi MI, Alqahtani AS, Al Aboud D, Abdel-Moneim AS (2021). BNT162b2 and ChAdOx1 SARS-CoV-2 post-vaccination side-effects among Saudi vaccinees. Front Med (Lausanne).

[47] Mallhi TH, Khan YH, Butt MH, Salman M, Tanveer N, Alotaibi NH (2022). Surveillance of side effects after two doses of COVID-19 vaccines among patients with comorbid conditions: a sub-cohort analysis from Saudi Arabia. Medicina (Kaunas).

[48] García-Araiza MG, Martinez-Cuazitl A, Cid-Dominguez BE, Uribe-Nieto R, Ortega-Portillo R, Almeyda-Farfán JA (2021). Side effects of the BNT162b2 vaccine in the personnel of the Military Central Hospital. Eur Rev Med Pharmacol Sci.

[49] Angyal A, Longet S, Moore SC, Payne RP, Harding A, Tipton T (2022). T-cell and antibody responses to first BNT162b2 vaccine dose in previously infected and SARS-CoV-2-naive UK health-care workers: a multicentre prospective cohort study. Lancet Microbe.

[50] Frieman M, Harris AD, Herati RS, Krammer F, Mantovani A, Rescigno M (2021). SARS-CoV-2 vaccines for all but a single dose for COVID-19 survivors. EBioMedicine.

[51] Powell AA, Power L, Westrop S, McOwat K, Campbell H, Simmons R (2021). Real-world data shows increased reactogenicity in adults after heterologous compared to homologous prime-boost COVID-19 vaccination, March–June 2021, England. Euro Surveill Bull = Europeen sur les maladies transmissibles European communicable disease bulletin.

[52] Mathioudakis AG, Ghrew M, Ustianowski A, Ahmad S, Borrow R, Papavasileiou LP (2021). Self-reported real-world safety and reactogenicity of COVID-19 vaccines: a vaccine recipient survey. Life (Basel, Switzerland).

